# In-person retail marketing claims in tobacco and E-cigarette shops in Southern California

**DOI:** 10.1186/s12971-017-0134-y

**Published:** 2017-06-17

**Authors:** Joshua S. Yang, Michele M. Wood, Katelynn Peirce

**Affiliations:** Department of Health Science, California State University, 800 N. State College Blvd., KHS 161A, Fullerton, CA 92834 USA

**Keywords:** E-cigarette, Marketing, Retail stores, Simulated customer

## Abstract

**Background:**

E-cigarette use has been increasing in the United States, though knowledge of potential risks and harms associated with e-cigarette use is low. Marketing of e-cigarettes may serve as a source of information to shape beliefs and attitudes toward e-cigarettes. The purpose of this study was to identify the most common marketing claims made within “vape” and tobacco shops in sales interactions with customers in demographically diverse cities.

**Methods:**

Vape and tobacco shops from three diverse cities in Southern California were selected for inclusion in the study. From May 2015 to July 2015, simulated customers asked salespeople in vape and tobacco shops how e-cigarettes compare to conventional cigarettes, and then recorded the resulting claims that were made using a standardized form designed for this purpose. Data were analyzed from January to March 2016.

**Results:**

The most frequent claims made by sales staff were that: smoking e-cigarettes helps one quit smoking (57% of the simulated shopping interactions), e-cigarettes come in multiple flavors (54%), and e-cigarettes are healthier than conventional cigarettes (50%). Simulated customer interactions that took place in vape shops included more positive marketing claims than those that occurred in tobacco shops; this relationship approached statistical significance (*p* = .087). There was a significant relationship between city and the average number of positive e-cigarette claims made (*p* < .001).

**Conclusions:**

A wide range of marketing claims are made about e-cigarettes in retail settings. These may vary by geographic location, community demographics, and type of retail outlet.

## Background

The U.S. Surgeon General has declared e-cigarette use among U.S. youth and young adults a major public health concern [1]. Electronic nicotine delivery systems (ENDS) are battery powered devices that use an electric charge to vaporize a solution made up of propylene glycol, and/or vegetable glycerin, flavorings, and may contain nicotine at varying concentration levels including no nicotine. Since their introduction in the United States in 2007, awareness and use of ENDS among U.S. adults has been increasing [2, 3]. Rates of ENDS use among youth have been increasing, as well, as they have become the most common form of tobacco used by middle school and high school students in 2014 [4], though recent data suggest vaping among teens may be beginning to decline [5].

The increase in ENDS use and projected growth in sales [6] has been accompanied by the emergence of vape shops that specialize in the sale of ENDS [7]. They differ from other outlets by providing a wider variety of products, selling newer generation devices, and allowing customers to sample ENDS products. In addition to selling ENDS, vape shops also play an important role in providing information [8, 9]. Vape shop owners and employees have positive attitudes towards and beliefs about ENDS [8–10] and engage in marketing which resembles tobacco industry marketing practices [11]. In addition to selling and providing information about ENDS, vape shops build rapport with customers and create an atmosphere around vaping which allows for interaction, builds a sense of community, and attracts customers [7, 9, 12, 13]. Though understanding of vape shops and their role in promoting ENDS is expanding, studies examining vape shop characteristics and dynamics across different racial/ethnic communities are still relatively uncommon [10, 14].

Few studies of vape shops examine employee-customer interactions. Information on interactions are based on self-reported attitudes, beliefs, and behaviors of vape shop owners and employees [8, 9]. One recent naturalistic observation study of employee-customer interactions in vape shops found 62% of customers had conversations while in the store, including small talk and about cloud chasing, products, and service requests [12]. Though awareness and use of e-cigarettes are increasing, knowledge of e-cigarette constituents has been shown to be low among young adults [15]. Thus, information about ENDS communicated in vape shops – and those specifically about the health and cessation qualities of ENDS – may serve as a source of information to shape beliefs and attitudes toward ENDS among potential users [8].

Exposure to ENDS marketing has been shown to be associated with incorrect knowledge about the presence of nicotine in e-cigarettes [15] and interest in e-cigarette trial [16], while high receptivity to e-cigarette marketing has also been associated with perceptions of e-cigarettes as less harmful than cigarettes, which is associated with higher recent e-cigarette use [17]. Thus, the purpose of the current study is to describe the marketing claims made by sales staff in vape shops located in demographically diverse communities using simulated customer methods. Specifically, a naturalistic study design was used to document characteristics of actual employee-customer interactions in vape shops to overcome validity concerns associated with retrospective self-reported data.

## Methods

### Context

This study used simulated customers inquiring about e-cigarettes at two types of shops (i.e., vape and tobacco) in three different cities in Orange County, CA to discover marketing claims made in different racial/ethnic communities. Rates of adult smoking in Orange County (10.8%) are below the Healthy People 2020 target and lower than the state adult smoking rate (11.6%) [18]. Yet recent data show that current and ever electronic cigarette use in Orange County middle school and high school students during the 2013–2014 school year was higher than use of conventional cigarettes, suggesting that ENDS are increasing in popularity [19].

Research assistants posed as shoppers, asked the salesperson how e-cigarettes compare to conventional cigarettes, and then recorded claims made by salespeople using a standardized form designed for this purpose. Informed consent was waived for simulated customer interactions. The use of deception in simulated customer studies allows for behaviors to be observed without changing the behavior because of the presence of an observer [20]. The gains in study validity with the use of deception by simulated customers could not be maintained if informed consent from individual participants was sought. Modified informed consent procedures for store-level consent to be included in the study may also bias study results. Under opt-out consent procedures, the validity of consent is questionable because lack of refusal may not reflect actual consent on the part of ENDS retailers but lack of attention to informed consent notices. Thus, given the minimal risk posed to human subjects involved in simulated customer interactions, anonymous collection of data, and the challenge to scientific and consent validity of alternate procedures, a waiver for obtaining informed consent for simulated customer interactions was requested and granted by the IRB. The research protocol was approved by the California State University Fullerton Institutional Review Board (HSR 15_0072, February 8, 2015). Simulated customer events (*N* = 68) took place from May 2015 to July 2015.

### Sample

*Shops.* For the purposes of this study, e-cigarette or “vape” shops were defined as retail outlets that sell e-cigarettes, e-cigarette components, and/or e-cigarette liquids, exclusively. Tobacco shops were defined as retail outlets for which conventional tobacco products (e.g., cigarettes, chewing tobacco) make up at least 50% of all products sold, and that also sell some type of electronic nicotine delivery device.

*Cities.* In Orange County, CA, the two largest racial/ethnic minority groups are Latinos (34.2%) and Asians (18.9%), with Mexicans the largest Latino subgroup in the county (29.3%; 85.8% of Latinos) and Vietnamese (6.3%; 33.1% of Asians) and Koreans (3.0%; 15.8% of Asians) two of the largest Asian ethnic groups in the county [21]. These three ethnic groups are important vulnerable populations for tobacco control; Koreans and Vietnamese smoke at higher rates than the general population and more than other Asian subgroups [22], and Latinos are the largest minority group in California.

Three cities were selected for inclusion in the study using purposive sampling, a type of non-probability sampling in which decisions about the elements to be included in the sample are made by the researcher based on a variety of criteria pertinent to the given study [23]. Specifically, the cities were chosen based on their historical and documented enclaves of Vietnamese, Korean, and Mexican populations [24, 25] and the substantially larger proportion of business serving them than in the general population. Based on 2015 U.S. Census data [21], 39.0% of the population in City A (174,721 total) was Asian, with 29.9% of the population identifying as Vietnamese and 2.8% as Korean. In City A, 36.7% of the population was Latino and 21% non-Hispanic White. Home to a two-mile stretch of Korean-owned businesses which attract Korean customers from the surrounding area [24], 52% of businesses in City A were Asian-owned in 2012. In City B, 48.2% of the population (91,719 total) was Asian, with 40.3% identifying as Vietnamese; 23.2% were Latino and 24.5% non-Hispanic White. A Vietnamese commercial district is centrally located in City B, but spreads to surrounding communities [24]. Of all businesses in City B, 54.2% were Asian-owned in 2012. In City C, 78.2% of the population (333,268 total) was Latino, with 72.6% identifying as Mexican; 10.6% were Asian and 9.2% non-Hispanic White. In City C, 31.8% of businesses were Hispanic-owned and 18.2% were Asian-owned in 2012. Thus, City A can be said to represent large Vietnamese and Korean populations; City B, a large Vietnamese population; and City C to represent a large Hispanic/Latino population.

*Sampling Frame.* To be included in this study, a retail outlet had to be either a vape or tobacco shop as defined above, and located within one of the three study cities. There is no definitive way of identifying ENDS retail outlets in the cities included in the sample. Thus, two methods were used to create a list of vape shops and tobacco shops located within the three study cities. First, Internet searches using the terms “vape shop,” “tobacco shop,” and “e-cigarettes” were conducted using Yelp and Google Maps websites, a method similar to online search strategies used in a previous study [14]. Second, a windshield survey of the three communities was conducted to verify stores identified through Yelp and Google Maps, visually identify vape and tobacco shops which may not have Yelp reviews or be listed through Google Maps, and confirm which tobacco shops sold ENDS. This was especially important to identify small tobacco shops which may not have social media following. Based on an established windshield survey methodology [26], researchers drove through each city to confirm whether stores identified through Yelp and Google Maps were still open and to identify vape or tobacco shops not found on Yelp or Google Maps. Once identified, researchers stopped at each business to determine whether the store met the inclusion criteria; if a store met the inclusion criteria, the store name, address, and whether it was a vape shop or a tobacco shop were recorded. Stores listed on Yelp or Google Maps that did not meet in the inclusion criteria or had closed down were not included in the sampling frame. The list of businesses created by the Internet search and windshield survey constituted the sampling frame; all shops identified through the two methods that met the inclusion criteria were included in the study.

*Sample Description.* A total of 68 retail outlets – 50 tobacco shops (74%) and 18 vape shops (26%) – were identified and included in the study. Of these, 44% were in City A (7 vape shops, 23 tobacco shops), 25% in City B (5 vape shops, 12 tobacco shops), and 31% in City C (6 vape shops, 15 tobacco shops). Just over half of the interactions (56%) involved female simulated customers, while 44% involved male simulated customers.

### Measurement

*Instrument.* The data collection instrument used to document simulated shopping experiences was developed based on marketing claims identified from content analysis of e-cigarette retailer websites [27] and Camel Snus magazine advertisements [28]. Marketing claims about the benefits of smoking e-cigarettes were listed in a table on the data collection form which, after the simulated customer interaction, the research assistant used to record the claims made by salespeople. In addition, the data collection instrument included field notes, where the research assistant could record other notable features of the interaction.

*Training.* Simulated customers were one graduate and five undergraduate student research assistants, ages 19–23 years, who received formal training for the study. The training covered the purpose of the study, study methods, review of the data collection instrument, and role play. The forms were pretested in shops outside the study area prior to use as part of field training. Research assistants were sent into four vape and tobacco shops in non-study cities, where they practiced engaging in simulated customer interactions and recording marketing claims using the data collection form. A debriefing training was held after the field training to identify emergent questions and unforeseen issues, and to streamline the data collection protocol.

*Variables.* Thirteen different claims made by salespeople during simulated customer interactions were recorded using the data collection instrument. Claims were preselected for inclusion in the study based on previous studies which examined marketing claims made by e-cigarette manufacturers and retailers [27, 28]. These included claims that e-cigarettes: 1) help one quit smoking, 2) are healthier, 3) can be used in more locations, 4) can be used anytime and anywhere, 5) do not generate second hand smoke, 6) are less expensive, 7) are friendlier to the environment, 8) are cleaner, 9) are more fire safe, 10) have no offensive odor, 11) are more socially acceptable, 12) are “cooler”, and 13) come in a wider variety of flavors, as compared to conventional cigarettes. In addition to these positive claims about the benefits of smoking e-cigarettes, corresponding negative claims also were recorded (i.e., e-cigarettes do *not* help on quit smoking, are *not* healthier, etc.). Thus, claims were recorded using 26 different variables, 13 representing positive claims and 13 representing corresponding negative claims. Simulated customers also maintained field notes on any discussions not captured by the thirteen different claims which occurred during simulated customer interactions or on the general nature of the interaction.

*Scaling.* The outcome for this study was the total number of positive claims about e-cigarettes made by the salesperson during the simulated shopping interaction. Each of the 13 positive claim variables was coded as “0” if the claim was not made, and “1” if the claim was made, during the interaction. A summative scale totaling the number of positive claims made was the outcome variable for this study, with a possible range of 0–13.

### Procedure

*Data collection.* For each of the three cities, two research assistants, one male and one female, were assigned to serve as simulated customers. The research assistants were bilingual in Korean for City A, bilingual in Vietnamese for City B, and bilingual in Spanish for City C. Within each city, the simulated customers were randomly assigned to the retail outlets included in the sampling frame. Simulated customer interactions took place on weekdays during business hours, between 9:00 am and 8:00 pm.

For each simulated shopping interaction, the research assistant entered the retail outlet and announced to a salesperson that he or she was interested in learning more about e-cigarettes. After making this introductory statement, the research assistant then asked the specific question, “Can you tell me more about e-cigarettes?” After the salesperson responded, the research assistant followed up with the question, “What is the difference between e-cigarettes and conventional cigarettes?” Once the salesperson responded, the research assistant thanked the salesperson and exited the shop. The research assistant then completed the data collection sheet to document which of the 13 marketing claims had been made, and which, if any, had been countered, by the salesperson.

*Analysis.* Data analysis was conducted from January 2016 to March 2016. Univariate analysis included frequency, mean, and standard deviation. Bivariate relationships were tested using independent samples *t*-tests and oneway analysis of variance (ANOVA). Post-hoc analysis was conducted using Tukey’s honest significance difference (HSD) test. Multivariate analysis consisted of multiple linear regression to examine factor**s** related to the number of marketing claims made; predictors included type of shop and city. A preliminary automated quantitative content analysis [29] of field notes from all simulated customer interactions was conducted using Atlas.ti 7.0 qualitative data analysis software [30]. Field notes were copied verbatim into a digital format and imported into Atlas.ti. The Word Cruncher function in Atlas.ti was used to create a frequency list of words mentioned in field notes. Words were ordered based on the frequency of mentions; common words such as “the”, “to”, and “and” were removed from the analysis.

## Results

Univariate analysis showed that the single most frequent claim about the benefits of smoking e-cigarettes made was that smoking e-cigarettes helps one quit smoking; this claim was made in 57% of the simulated shopping interactions. In just over half of the interactions (54%), the salesperson made the claim that a benefit of e-cigarettes is that they come in multiple flavors, and in half the interactions (50%), the salesperson claimed that e-cigarettes are healthier than conventional cigarettes. All claims were made at least once. (See Table 1 for frequencies of all positive and negative claims made.) Field notes were made in 55 simulated customer interactions. Quantitative content analysis of field notes showed that the most frequently recorded word not captured among the 13 predetermined marketing claims was “nicotine” which was mentioned in 18 simulated customer interactions, most often related to the ability to adjust the level of nicotine consumed when using e-cigarettes.

**Table 1:** Percentage of simulated customer interactions in which e-cigarette marketing claims were made (N = 68)

	% of Interactions with Statements	
E-Cigarette Claim	In Agreement	In Disagreement
Help you quit smoking	57	3
Come in flavors	54	0
Healthier	50	2
Cleaner	44	2
Less expensive	37	2
Can be used in more places	21	0
Can be used anytime/anywhere	19	0
No offensive odor	10	0
“Cool” factor	10	0
More socially acceptable	9	0
Do not generate 2nd hand smoke	6	0
Friendlier to environment	6	0
More fire-safe	3	0

**Fig. 1:**
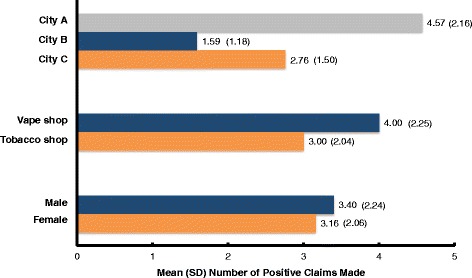
Average number of positive claims made about e-cigarettes by city, shop type, and sex (*N* = 68). Shows the average number of marketing claims made by city, type of shop, and whether the simulated customer was male or female. Statistically significant differences in mean number of claims made were found by city and shop. More claims were made in the Korean-dominant city compared to Vietnamese- and Latino-dominant cities, and more claims were made in vape shops compared to tobacco shops. No difference was found between male and female simulated customers

Bivariate analysis compared the number of positive claims made about e-cigarettes by shop type, city, and sex. (See Fig. 1.) Simulated customer interactions that took place in vape shops included more positive marketing claims (*M* = 4.00, *SD* = 2.25) than those that occurred in tobacco shops (*M* = 3.00, *SD* = 2.04); this relationship approached statistical significance, *t*(66) = −1.74, *p* = .087. There was a significant relationship between city and the average number of positive e-cigarette claims made, *F*(2,65) = 16.95, *p* < .001. Simulated customer interactions that took place in City A included significantly more positive marketing claims by sales staff (*M* = 4.57, *SD* = 2.16) than did interactions in City B (*M* = 1.59, *SD* = 1.50, *p* < .001) and City C (*M* = 2.76, *SD* = 1.18, *p* = .002). Sex of the simulated customer was not associated with number of positive claims made, *t*(66) = −.463, *p =* .645.

Multiple regression analysis was used to test whether shop type and location were significant predictors of the number of positive e-cigarette marketing claims made. Together, shop type and city accounted for 38% of the variance in number of positive claims made about e-cigarettes (adjusted *R* ^*2*^ *= .38, F*(3,64) = 14.40, *p* < .001). Type of shop was a significant predictor controlling on city, with vape shops being associated with a larger number of marketing claims than tobacco shops (*β* = .25, *p* = .013). City also was a significant predictor, with City A associated with significantly more marketing claims than both City B (*β* = −.62, *p* < .001) and City C (*β* = −.41, *p* < .001), controlling for shop type. (See Table 2.)

**Table 2:** Number of positive e-cigarette marketing claims regressed on shop type and city (N = 68)

Variable	B	SE(B)	Beta	t	*p*
Constant	4.29	.33		13.18	<.001
Vape shop	1.18	.46	.25	2.54	.013
City B	−3.05	.51	−.62	−5.96	<.001
City C	−1.87	.48	−.41	−3.90	<.001

Adjusted *R* ^2^ = 38%, *F* = 14.40, *p* < .001

## Discussion

The results of this study show that a wide range of marketing claims are made about e-cigarettes in retail settings. Two of the most frequently made claims suggested e-cigarettes could help smokers quit and that they were healthier than conventional cigarettes. Using a novel methodology, these findings align with previous studies which suggest that vape store owners and employees believe in the benefits and safety of e-cigarettes compared to conventional cigarettes, adding to and confirming the literature that they communicate such beliefs in customer interactions. For example, Wagener et al. [13] found that the two most common reasons cited for starting to vape among vape shop customers was to stop/reduce smoking and to improve health, which align with the two of the three most common marketing claims in the present study. Similarly, 80% of vape shop customers use and prefer non-tobacco flavored e-liquid, reflecting the importance of flavors in marketing claims made by sales people in our findings. The wide range of claims made about e-cigarettes also suggests that much unsubstantiated information is being presented by retailers to customers.

Bivariate analysis suggests that the number of claims made about e-cigarettes vary by store type and by city. Employees of vape shops made more claims about e-cigarettes than did those of tobacco shops. This is likely due to the specialized nature of vape shops and the high level of investment they have in e-cigarettes. Though tobacco shops carry e-cigarettes, they tend to be one of a wider variety of tobacco (e.g., conventional cigarettes, water pipe glass, smokeless tobacco) products. Engagement with retailers on regulation of e-cigarettes should take into consideration the positioning of e-cigarette products within a store’s product portfolio and overall business model. Variation in claims made by city suggest that exposure to claims about e-cigarettes may depend on where customers purchase their products. Multivariate analysis found that the type of retail shop (i.e., vape or tobacco) and the city in which the shop was located were both significant independent predictors of the number of positive claims made about e-cigarettes. Significantly more positive marketing claims were made in vape shops than in tobacco shops, while holding city constant, and significantly more claims were made in City A, relative to Cities B and C, holding shop type constant. City A is a more ethnically heterogeneous than the two other cities, and we speculate that this may be a factor in explaining difference by city. The relationship between ethnic composition of and marketing claims made in cities should be explored in future research.

Multivariate analysis confirmed bivariate findings; thus, the difference in the number of positive marketing claims made by city was not merely a function of the proportion of vape shops included. Together, the type of shop and the city in which it was located accounted for a large proportion (38%) of the variance in the number of positive marketing claims made about e-cigarettes. These findings suggest that to be most effective, interventions should be aware of patterns that may exist in the types of retail businesses and that communities with a large number of vape shops should be prioritized. Furthermore, although we found that e-cigarette marketing claims were made in all three communities we studied, the number of claims, and thus the degree of marketing influence, varied across ethnically diverse communities. Education and countermarketing campaigns can improve their effectiveness by developing programs that take into account the local context and specific needs of the communities they target.

U.S. Food and Drug Administration regulation of e-cigarette and ENDS sales includes minimum sale age requirements and warning labels on all e-cigarette or ENDS products and advertising. Regulations, however, do not affect what retailers can say to customers in in-person interactions. A strong countermarketing campaign is needed to ensure evidence-based information about their safety and the efficacy of ENDS as cessation devices is reaching potential adopters and existing customers in the retail setting. Such engagement should consider local variation in shop types and distribution to best target outreach efforts. Similarly, education of vape shops owners may be an area of future intervention to minimize potential harm caused by misinformation conveyed by vape shop employees.

### Strengths and limitations

The methods used in this study are a strength. Compared to previous studies, our method of data collection allowed us to more closely capture the experience of e-cigarette shoppers. The replication of previous findings using a different and more “real world” approach provides support for the validity of prior research. This study included retail outlets serving underrepresented and understudied communities. The results provide better understanding of information being communicated in ethnic minority communities, and suggest ENDS marketing may have community-specific characteristics that should be taken into consideration in future educational and countermarketing interventions.

The present study has several limitations. The sample size limits the ability to conduct more detailed analysis and may mask additional patterns in marketing claims. There may be recall bias in recording marketing claims after simulated customer interactions, though the time between simulated customer interactions and recording of claims was kept to a minimum, and would be expected to introduce much less bias than self-reporting of prior events. The study was limited to a small geographic area which may limit generalizability to other cities or geographic areas. The average age of simulated customers was lower than the reported average age of customers to vape shops [12], which may also limit generalizability of results.

Future research should be expanded beyond specialty shops to examine marketing claims across the breadth of retail channels where e-cigarettes are available and the reception of those claims by potential customers. Segmenting receptivity to marketing claims by potential user groups is also needed. Additionally, geospatial analysis of e-cigarette marketing claims and sales can provide needed information on the clustering of information channels for targeting of outreach efforts and countermarketing.

## Conclusions

Vape and tobacco shops are an important source of information about e-cigarettes for consumers. A wide variety of marketing claims are made about e-cigarettes in retail settings, the most frequent relating to the health benefits of e-cigarettes and their efficacy for smoking cessation. Public health efforts to provide accurate information on e-cigarettes should consider actions e-cigarette retailers take as conduits of information for potential users and attuned to which claims are being made in order to prioritize educational and countermarketing messaging.

## Abbreviation

ENDS: Electronic nicotine delivery system
